# White Blood Count Can Be a Practical Guide for the Differential Diagnosis of Breast Abscess and Idiopathic Granulomatous Mastitis

**DOI:** 10.7759/cureus.10468

**Published:** 2020-09-15

**Authors:** Çağrı Akalın, Hilal Altaş, Mürüvvet Akçay Çelik

**Affiliations:** 1 General Surgery, Ordu University, Training and Research Hospital, Ordu, TUR; 2 Radiology, Ordu University, Faculty of Medicine, Ordu, TUR; 3 Pathology, Ordu University, Faculty of Medicine, Ordu, TUR

**Keywords:** idiopathic granulomatous mastitis, breast abscess, neutrophil to lymphocyte ratio, white blood cells, c-reactive protein

## Abstract

Introduction and aim

Idiopathic granulomatous mastitis (IGM) is an inflammatory disease of the breast and has the same symptoms and radiologic imaging as breast abscess (BA). The aim of this study is to evaluate the use of inflammatory markers as white blood count (WBC), C-reactive protein (CRP), and neutrophil to lymphocyte ratio (NLR) as a potentially useful tool for the differential diagnosis of BA and IGM.

Methods

In this retrospective study, we analyzed 31 patients with IGM and 47 patients with BA between January 2013 and April 2020. Age, symptoms, symptomatic breast side, microbiological culture, complete blood count, and C-reactive protein (CRP) values of patients were analyzed. Receiver operating characteristic (ROC) curve analysis was used to define the optimal cut-off for WBC, CRP, and NLR.

Results

WBC was significantly higher in the BA group compared to the IGM group (11.45 vs. 9.78; p=0.042), but no difference was found for CRP and NLR between these groups (p=0.146, p=0.081, respectively). In ROC analysis results in BA group, cut-off values, the best sensitivity and specificity for WBC, CRP, and NLR were 8.46 × 10^3^/μL (81%-70%), 1.5 mg/dl (77-76%), and 2.93 (70-82%), respectively. For IGM group, cut-off values, the best sensitivity and specificity for WBC, CRP and NLR were 8.49 × 10^3^/μL (74-70%), 1.5 mg/dl (61-76%) and 2.29 (64-72%), respectively.

Conclusion

This study showed that CRP and NLR cannot be used as a useful tool for differential diagnosis of IGM; furthermore, WBC is a parameter that can act as a practical guide for the differential diagnosis of BA and IGM.

## Introduction

Idiopathic granulomatous mastitis (IGM) is a chronic, inflammatory disease of the breast defined in 1972 by Kessler and Wolloch. Its clinic and radiologic findings may be confused with breast cancer and breast abscess (BA) [1]. The diagnosis is made by excluding the reasons causing granulomatous mastitis (GM), in addition to histopathological examination [2].

In cases of systemic inflammation and infection, the traditional inflammatory mediators, such as white blood cell (WBC), C-reactive protein (CRP), and acute-phase proteins, are used for diagnosis and follow-up [3]. Neutrophil to lymphocyte ratio (NLR) is a new marker obtained from peripheral blood analysis, which is cheap, non-invasive, and calculated easily. NLR has been used as a diagnostic marker recently for infections accompanied by systemic inflammation [4]. In the literature, there are a limited number of studies investigating the relationship between IGM and inflammatory markers, and these studies focused specifically on the recurrence of IGM [5, 6]. Unfortunately, there are no studies about diagnostic tools that can be used for the differential diagnosis of IGM and BA.

The aim of this study is to evaluate the use of inflammatory markers as WBC, CRP, and NLR as a practical guide for the differential diagnosis of BA and IGM.

## Materials and methods

This was a retrospective case-control study approved by the clinical research ethics committee of the institution. We enrolled 31 patients with IGM and 47 patients with BA at a high-volume single-center hospital between January 2013 and April 2020. The patients were analyzed in terms of age, symptoms, symptomatic breast side, microbiological culture, complete blood count (CBC), and C-reactive protein (CRP) values. The exclusion criteria for the study were: 1) being under 18, 2) pregnancy, 3) having chronic diseases (diabetes mellitus, hypertension, inflammatory bowel disease, hematologic diseases, etc.) or using drugs (intravenous immunoglobulin, thyromazol, etc.) which may affect NLR, 4) malignancy, 5) patients who had microbiologic factors (mycobacterium tuberculosis, blastomycosis, Corynebacterium, etc.), autoimmune diseases (Wegener granulomatosis, giant cell arteritis, foreign body reaction), ductal ecstasy (plasma cell mastitis, subareolar granuloma), sarcoidosis, fat necrosis, which cause GM. Patients data such as clinic information, laboratory parameters and microbiological culture was accessed from the hospital data system and patient files. Additionally, information on the history of chronic diseases, malignancy, and the use of drugs was analyzed from the National Personal Health System (e-pulse).

IGM patients consisted of undiagnosed patients whose histopathological result was GM. Patients with BA consisted of those with an abscess which was diagnosed by physical examination and/or ultrasound (US), with growth in microbiology culture and who responded to drainage w/o antibiotic treatment. The control group was selected amongst healthy volunteers referred to the general surgery clinics of our hospital for a health check-up.

The differential diagnosis of GM was done with the evaluation of patients by physicians in the existing hospital. Etiologies to consider in the differential diagnosis of GM included the following: microbiological analysis (bacteriologic culture, fungal stains, and acid-fast bacilli for tuberculosis), laboratory tests (anti-double-stranded DNA, anti-nuclear antibody, anti-neutrophil cytoplasmic antibodies, rheumatoid factor), and purified protein derivative skin test. The abscess drainage, biopsy, and histopathologic examinations were performed by the specialist physicians (general surgery, radiology, and pathology) at the same hospital. Drainage of the abscess was performed with US-guided needle aspiration or incision for BA. Breast biopsies were taken as a core biopsy for IGM. On the histopathological examination, observation of non-caseating granuloma, epithelioid histocytes, and lymphocyte, neutrophils, and eosinophils, with a spread in the perilobar region was assessed in favor of IGM.

The patients’ CBC and CRP results were obtained and analyzed at the time of the first admission to the outpatient clinic. For CBC, venous blood samples were taken in the tubes containing ethylenediaminetetraacetic acid and analyzed on the automatic blood analysis device (Sysmex XN-1000™, Kobe, Japan) in the hematology laboratory. For CRP, venous blood samples were collected in empty tubes and analyzed on the Cobas® 6000 autoanalyzer (Roche, Mannheim, Germany). The CBC and CRP values were assessed according to the reference interval accepted by the hematology laboratories nationwide. WBC, neutrophil, and lymphocyte values were obtained from the CBC analysis. NLR was calculated by dividing the neutrophil count by the lymphocyte count.

Data were analyzed using Statistical Package for Social Sciences (SPSS, version 25.0, IBM Inc., Armonk, USA). Descriptive statistics for continuous variables are expressed as mean, standard deviation, minimum and maximum. Categorical variables are expressed as number and percentage. The chi-square test was used to determine the relation between categorical variables. The data distribution was evaluated using the Kolmogorov-Smirnov test. Mann-Whitney U test and Kruskal-Wallis test were performed for continuous variables. Receiver operating characteristic (ROC) curve analysis was used to define the optimal cut-off for WBC, CRP and NLR. P value <0.05 was considered statistically significant.

## Results

In the study, there was a total of 128 participants - 47 (36.7%) in the BA group, 31 (24.2%) in the IGM group, and 50 (39.1%) in the control group. The mean age of patients was 38.41±9.68 years (min 19 - max 64). There was no significant difference between groups in terms of age (p=0.67). While the breast lesions were observed in the right breast in 22 patients (46.8%) and the left in 25 (53.2%) of 47 patients with BA; they were observed in the right breast in 13 patients (41.9%) and left in 18 (58.1%) out of 31 patients with IGM. On physical examination, the pain was observed in all of the patients with BA (n=47; 100%) with swelling in 38 (80.8%), rush in 31 (65.9%), and axillary lymphadenopathy in 21 (44.7%). For IGM patients, the pain was observed in 25 of the patients (n=31; 80.6%), swelling in 22 (70.9%), rush in 18 (58.1%), and axillary lymphadenopathy in seven (22.6%). There was no significant difference between BA and IGM in terms of symptoms and symptomatic breast side (p=0.67, p=0.37, respectively).

While abscess was found on US in four (12.9%) out of the 31 patients with IGM, no growth was found in the microbiological culture. The US results of 42 (89.4%) patients with BA were found to be consistent with the abscess; there was no US analysis for the remaining five (10.6%). Considering the microbiological culture results of the patients with BA, Staphylococcus aureus was found in 31 (66%) out of 47 patients, Staphylococcus epidermidis in eight (19.6%), Streptococcus pyogenes in three (7.3%), Staphylococcus aureus + Anaerobic cocci in two (4.9%), Escherichia coli in one (2.4%), Staphylococcus aureus + Escherichia coli in one (2.4%), and Bacteroides spp. in one (2.4%) patient.

WBC, CRP, and NLR values were significantly higher in BA and IGM groups compared with the control group (for all parameters p<0.001). Furthermore, when BA and IGM groups were compared, while a significant difference was found in WBC between these groups (p=0.042), no difference was found for CRP and NLR (p=0.146, p=0.081, respectively). The laboratory data of all groups are given in Table 1.

**Table 1:** Laboratory data from all groups WBC - white blood count; CRP - C-reactive protein; NLR - neutrophil to lymphocyte ratio; BA - breast abscess; IGM -  idiopathic granulomatous mastitis p<0.05 is considered to be significant

Variables	Control group (n=50) (mean ± sd, min-max)	BA group (n=47) (mean ± sd, min-max)	IGM group (n=31) (mean ± sd, min-max)	p
WBC (10^3^/μL)	7.29±1.89 (3.95-11.41)	11.45±3.44 (5.82-22.4)	9.78±2.74 (5.02-15.23)	<0.001
CRP (mg/dl)	1.04±0.98 (0.10-4.35)	3.40±3.27 (0.30-17.40)	2.29±1.69 (0.10-6.70)	<0.001
NLR	2.11 (0.66-4.65)	7.68±7.51 (0.79-29.35)	4.25±3.16 (1.08-15.23)	<0.001

**Figure 1:**
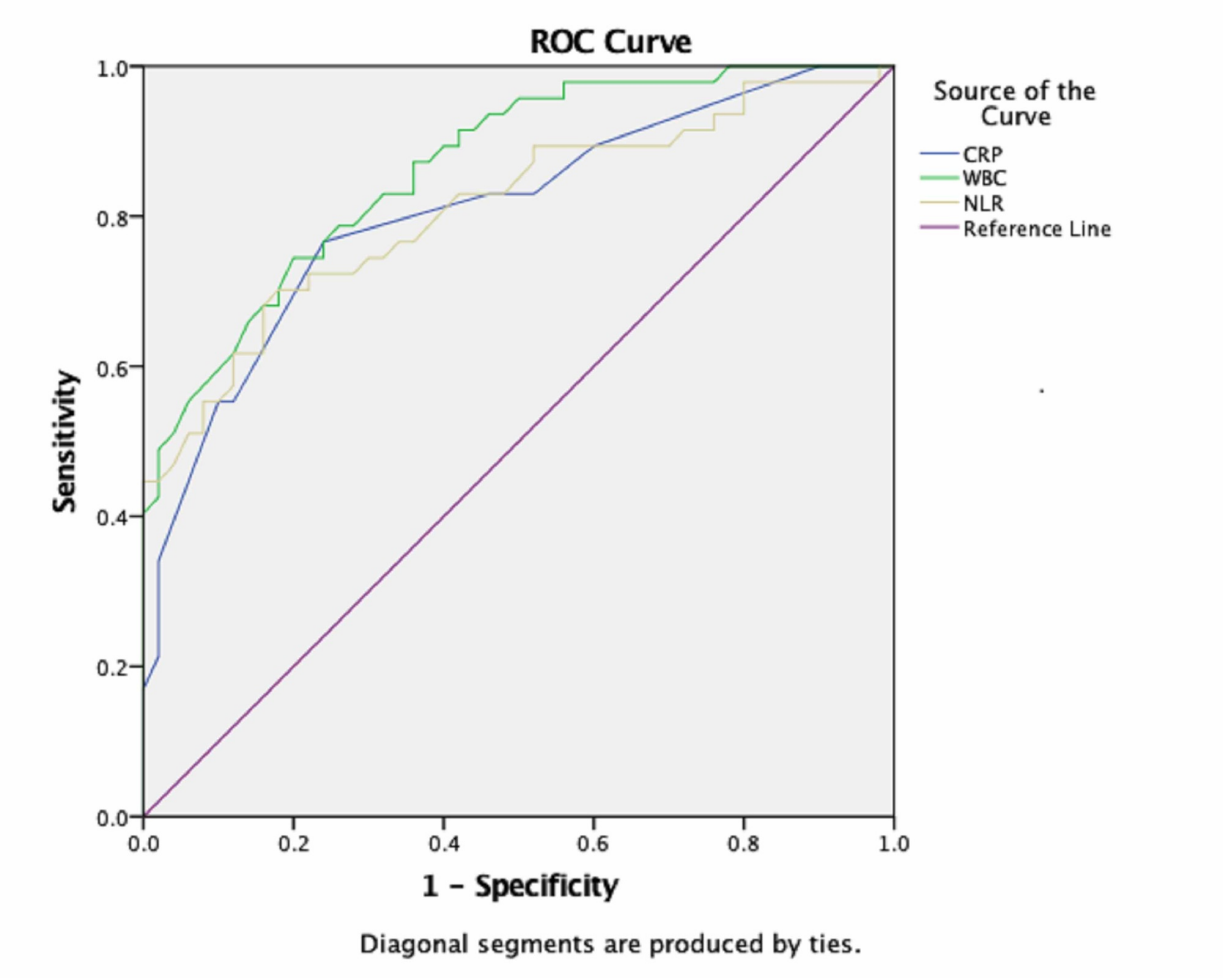
Receiver operating characteristic curve used to distinguish patients with BA from the control group WBC - white blood count; CRP - C-reactive protein; NLR - neutrophil to lymphocyte ratio; ROC - receiver operating characteristic; BA - breast abscess

**Figure 2:**
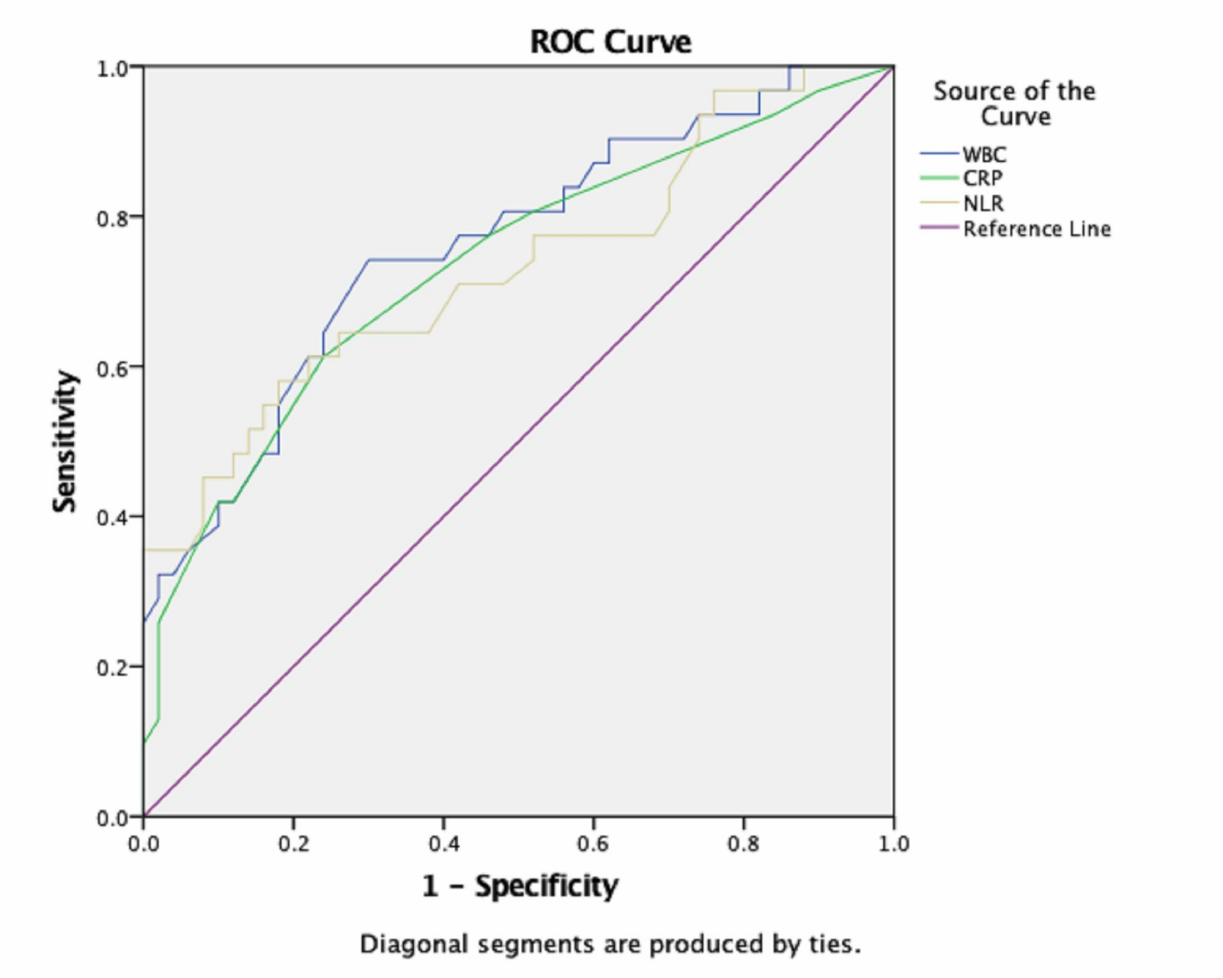
Receiver operating characteristic curve used to distinguish patients with IGM from the control group WBC - white blood count; CRP - C-reactive protein; NLR - neutrophil to lymphocyte ratio; ROC - receiver operating characteristic; IGM - idiopathic granulomatous mastitis

As a result of ROC analysis, there was no significant difference in WBC, CRP, and NLR between the BA and IGM groups (for all parameters p<0.001). ROC curves, containing the WBC, CRP, and NLR data for BA and IGM groups, are given in Figures 1 and 2.

In ROC analysis results in BA group, cut-off values, the best sensitivity and specificity for WBC, CRP, and NLR were 8.46 × 103/μL (81%-70%), 1.5 (77%-76%), and 2.93 (70%-82%), respectively. In ROC analysis results in IGM group, cut-off values, the best sensitivity and specificity for WBC, CRP, and NLR were 8.49 × 103/μL (74%-70%), 1.5 (61%-76%), and 2.29 (64%-72%), respectively. With respect to predicting a diagnosis of BA and IGM with ROC curve analysis, the area under curve (AUC) values for WBC, CRP, and NLR values and the other data are shown in Table 2.

**Table 2:** The area under the ROC curve of each marker WBC - white blood count; CRP - C-reactive protein; NLR - neutrophil to lymphocyte ratio; BA - breast abscess; IGM -  idiopathic granulomatous mastitis; ROC - receiver operating characteristic; AUC - area under curve; SE - sensitivity p<0.05 is considered to be significant

Variables	AUC	SE	p	95% Confidence Interval	
				Lower bound	Upper bound
WBC (10^3^/μL)					
BA	0.865	0.035	<0.001	0.796	0.935
IGM	0.761	0.056	<0.001	0.652	0.870
CRP (mg/dl)					
BA	0.808	0.044	<0.001	0.721	0.895
IGM	0.734	0.059	<0.001	0.618	0.850
NLR					
BA	0.811	0.044	<0.001	0.724	0.898
IGM	0.731	0.060	<0.001	0.613	0.849

## Discussion

The effect mechanism for IGM is that milk protein starts an autoimmune process characterized by non-caseating granuloma in the hypertrophic and fragile breast tissue [7]. Trauma, hormonal and metabolic disorders, oral contraceptive use, hyperprolactinemia, and bacterial factors like Corynebacterium are presented as the reasons which may give rise to this autoimmune process [8-11]. The real prevalence of IGM isn’t known yet, and it was stated that it is seen more in people of Asian and Latin American origin [12]. In our study, all of the patients lived in Turkey and had no significant distribution geographically. BA is defined as the collection in breast tissue characterized by purulent material [13]. Even though the most common cause of BA is Staphylococcus aureus, other bacterial, fungal, and granulomatous causes are also included in the etiology [14]. In the current study, Staphylococcus aureus was detected in most of the patients, and no fungus and Corynebacterium, which is considered to play a role in the IGM etiology, was found in any patient.

Recently, researchers investigated the association between inflammatory markers and abscess-forming diseases. In a study investigating the relationship between NLR and pancreatitis, Kaplan et al. found that WBC and CRP were significantly high in the pancreatic abscess. They also indicated that NLR could be used as a supportive indicator for the diagnosis of pancreatitis [15]. Similarly, Yildirim et al. reported that WBC and NLR levels were significantly high in tubo-ovarian abscess [16]. In addition, the NLR value >4.15 had 95.2% sensitivity and 99.4% specificity for the diagnosis of a tubo-ovarian abscess. In the study performed by Senturk et al., NLR was significantly higher compared with the control group before treatment in patients with peritonsillar abscess [17]. Moreover, it was stated by the authors that both the sensitivity and specificity were 90.9% at a cut-off value of 3.08 for NLR in the diagnosis of peritonsillar abscess. The advantage of our study, apart from these studies, is that we identified the bacterial microorganism causing BA with microbiological culture. In these studies, despite the relationship between abscess formation and high WBC, CRP, and NLR values, the sensitivity and specificity ratio of NLR in the BA group was determined to be lower. We consider that this difference may be caused by the variety of the microorganisms causing abscess in different anatomic localizations.

In recent years, NLR was used as a supportive marker for the diagnosis of inflammatory and autoimmune diseases [18, 19]. There are a limited number of studies that explore the association between NLR and IGM. In 2020, Çetinkaya et al. examined the predictive value of NLR and platelet lymphocyte ratio (PLR) in patients with recurrent IGM [5]. They found that the use of NLR seems to be cost-effective and is a promising indicator for the prognosis and recurrence of IGM. In the study by these authors, NLR and PLR were evaluated during the preoperative and postoperative period, whereas in our study, these parameters were evaluated at the beginning of diagnostic workup for IGM. Similarly, Kargın et al. found that pre-treatment NLR may provide an idea about predicting long-term recurrence after treatment in patients with GM [6]. In this study, the authors evaluated all patients with GM. However, we evaluated only patients with IGM in the current study. Generally, in named studies, NLR was shown to be a predictive factor for the recurrence of IGM. In contrast, in our study, both inflammatory markers were evaluated before the diagnosis of GM and compared between IGM and BA.

The findings in this study are subject to at least three limitations. First, it is a retrospective study. Second, we could not compare inflammation markers, such as tumor necrosis factor, interleukin-1β, and procalcitonin. Third, the patient groups are of a limited number. Despite these limitations, our study also has some advantages. First, we could not find any studies similar to ours in literature, and there are a limited number of studies on IGM, and these studies focus more on predicting relapse. Second, we think that including the control group in the study gives the advantage of comparing these diseases with healthy individuals. In the present study, the inflammatory markers in BA and IGM, which are two conditions that are very similar to each other, were examined. In line with our findings, these markers were evaluated as a diagnostic tool that can assist the clinician for both BA and IGM diagnosis, and we believe that our study will contribute to the literature.

## Conclusions

In the diagnosis of IGM, the differential diagnosis must include other granulomatous diseases and it is a big effort and occurs over a long period of time by clinicians. IGM mimics BA with its clinical and radiological conditions. This study indicates that CRP and NLR cannot be a useful tool for the differential diagnosis of BA and IGM. On the other hand, WBC is the parameter which may act as a much more practical guide for the differential diagnosis of these diseases.
